# Educational inequalities in self-rated health and their mediators in late adulthood: Comparison of China and Japan

**DOI:** 10.1371/journal.pone.0291661

**Published:** 2023-09-15

**Authors:** Ruru Ping, Takashi Oshio

**Affiliations:** 1 Graduate School of Economics, Hitotsubashi University, Tokyo, Japan; 2 Institute of Economic Research, Hitotsubashi University, Tokyo, Japan; Bar-Ilan University, ISRAEL

## Abstract

Education has an impact on health, but the magnitude of the impact may vary across countries. This cross-sectional study compared educational inequalities in health and their mediators in late adulthood between China and Japan, which both face rapid population aging. We studied the same age cohort (63–72 years) based on two nationwide population-based surveys in 2018: the China Health and Retirement Longitudinal Study (*N* = 5,277) and Japan’s Longitudinal Survey of Middle-Age and Elderly Persons (*N* = 20,001). The relative index of inequality (RII) in education was used to measure educational inequality in self-rated health (SRH). We then examined the extent to which income, smoking, leisure-time physical activity, and social participation mediated educational inequalities in SRH. In both countries, a lower educational level was associated with a higher risk of poor SRH; in China, however, the gradient was flatter. In China, the RII of education was 1.69 (95% confidence interval [CI]: 1.20–2.39) for men and 1.47 (95% CI: 1.06–2.05) for women. In Japan, meanwhile, RII was 2.70 (95% CI: 2.21–3.28) for men and 2.60 (95% CI: 2.13–3.18) for women. Our mediation analysis based on logistic regression models with bootstrapping also found that social participation was a key mediator of educational inequalities in health in both countries. In all, the results underscore that one’s relative position in educational inequalities is a reliable predictor of subjective health in late adulthood in both China and Japan.

## Introduction

Studies have shown a close relationship between education and health. For example, Conti et al. highlighted a causal effect in explaining differences in many adult outcomes and healthy behaviors, while emphasizing the heterogeneity [1]. Davies et al. showed that remaining in school causally reduced some health risks and mortality but highlighted genetic and socioeconomic confounding factors [2].

However, the relationship between education and health may be affected by historical socio-political contexts [3]. Specifically, the relative size of the effect of education on health may vary across countries due to different educational systems and their sociocultural implications. From a life course perspective, the level of education a person achieves reflects the far-reaching impact of their parents’ socioeconomic status and broader socioeconomic context on their socioeconomic status in young adulthood [4]. Education is also a strong predictor of important resources in adulthood, such as income and occupation [4].

This study compared educational gradients in self-rated health (SRH) in late adulthood in China and Japan, two countries that face an unprecedented pace of population aging [5]. We focused on cohorts aged 63 to 72 years old in 2018 (born between 1946 and 1955) in China and Japan. These cohorts shared similar life trajectories characterized by postwar construction, phenomenal economic growth, a rapid decline in total fertility rate, as well as reductions in child and adult mortality rates, leading to today’s ageing societies. The life experiences of our study cohorts differ from those developed in Western ageing societies, and they may affect how inequalities in educational attainment influence health status in late adulthood. This assertion is supported by a cross-sectional WHO study that identified socioeconomic inequalities in mental disorders in North American and European countries but not in emerging economies [6]. Despite their shared histories, China and Japan have distinct educational systems, with educational attainment in Japan generally being much higher than in China. This structural variation allows us to investigate the relative strength of the relationship between educational inequalities and healthy ageing.

Meanwhile, an increasing number of studies have focused on the underlying mechanisms linking education and health [7, 8]. Socioeconomic status (e.g., occupational status, income), health behaviors (e.g., smoking, physical activity, obesity), and social participation (e.g., volunteer work, community activities) are often considered potential mediators. However, the results have generally been mixed, partly because of the choice of health outcomes, mediator variables, and targeted age groups.

Several single-country studies have examined educational inequalities in health outcomes and health behaviors in China [9, 10] and Japan [11, 12]. Although some cross-country studies have compared Japan and Korea [13] and China and England [14], China and Japan have not yet been compared. Two recent comparative studies examined the relationship between socio-economic status and health across a range of countries, including China and Japan, but they did not investigate the mediators of educational inequalities in health [15, 16]. To our knowledge, this is the first study to compare educational inequalities in health outcomes in later life in China and Japan and examine the potential mediators underlying them.

Building on the existing literature, we hypothesized that a lower educational level would be associated with a higher risk of poor health status in late adulthood in China and Japan. However, the association may be weaker in Japan than China, due to potentially more prominent inequality in educational attainment among the birth cohort in China. All the socioeconomic and health behaviors mediators of interest could play a significant role in this association.

## Materials and methods

### Data and sample

We used data from two nationwide population-based social surveys that targeted middle-aged and older adults in China and Japan: the China Health and Retirement Longitudinal Study (CHARLS) conducted by Peking University and the Longitudinal Survey of Middle-Age and Elderly Persons (LSMAEP) conducted by the Japanese Ministry of Health, Labor, and Welfare (MHLW). The baseline CHARLS survey was conducted in 2011 (response rate: 80.5%) using multistage probability sampling to select 17,500 individuals aged 45 years or above from 150 counties across China. Follow-up surveys were conducted in 2013, 2015, and 2018. The LSMAEP has been conducted annually since 2005. The first wave comprised a cohort of participants aged 50–59 years. Samples for the first wave were collected from individuals nationwide in November 2005 using a two-stage random sampling procedure. In total, 34,240 individuals responded to the survey (response rate: 83.8%). The second through fourteenth waves were conducted in early November of each year, from 2006 to 2018.

We focused on the common cohort aged 63–72 in 2018 (born between 1946 and 1955) in China and Japan, whose data were collected from the CHARLS and LSMAEP surveys conducted that year. These surveys provided the most recent data available for comparing China and Japan at the time of this writing. The attrition rate from the baseline survey to the study survey was 29.4% for CHARLS and 38.3% for LSMAEP, although new participants were added in each CHARLS follow-up survey.

After excluding those who did not report any key variables, the sample consisted of 5,277 Chinese and 20,001 Japanese individuals. The authors had no access to information that could identify individual participants during or after data collection.

The CHARLS study protocol was approved by the Ethical Review Committee of Peking University. Data were obtained with Peking University’s official approval. Therefore, the current study did not require additional ethical approval. The committee waived the requirement for written consent. The LSMAEP was approved by Japan’s Statistics Act, which required it to be reviewed from statistical, legal, ethical, and other aspects. Data were obtained from MHLW with official permission. Therefore, this study did not require ethical approval. The need for written consent was waived in line with the Statistics Act.

### Measures

#### Educational attainment

In China, CHARLS asked respondents to select their educational attainment from among (1) illiterate, (2) literate but did not finish primary school, (3) *sishu* or homeschool, (4) elementary school, (5) junior high school, (6) high school, (7) vocational school, (8) two- or three-year college or associate degree, (9) four-year college or bachelor’s degree, (10) master’s degree, and (11) doctoral degree. These 11 categories were classified into five educational levels: illiterate (1), did not finish primary education (2), finished primary education (3–4), junior high school (5), and high school and above (6–11). In Japan, educational attainment was classified into six categories: (1) junior high school, (2) high school, (3) junior college, (4) college, (5) graduate school, and (6) other. Out of the entire sample, 119 respondents (0.6% of the total) reported their final educational attainment as “other” and they were excluded from the analysis in Japan.

We then ordered educational levels in ascending order from lowest to highest and calculated the ridit scores at each level as the proportion of individuals with lower educational level(s) plus one-half of the proportion of individuals in the category itself separately for China and Japan [17]. The ridit scores, which are designed to capture the relative position of each individual’s educational attainment compared to their peers, made it possible to compare the impact of educational levels which are classified in different ways between the two countries. In the regression analysis, ridit scores were used as continuous variables. We calculated the educational ridit scores for men and women separately for both countries, considering the substantial sex-based differences in educational attainment.

#### Health outcomes

We focused on self-rated health (SRH) because it is a well-recognized, comprehensive, and reliable indicator of general health conditions [18]. The CHARLS survey asked respondents to choose from “very good,” “good,” “fair,” “poor,” and “very poor” in response to the question: “What is the current condition of your health?” We constructed a binary variable for poor SRH by allocating 1 to the last two and 0 to the others. For SRH, the LSMAEP asked respondents to choose from “very good,” “good,” “somewhat good,” “somewhat poor,” “poor,” and “very poor”. We constructed a binary variable for poor SRH by allocating 1 to the last three and 0 to the others.

To assess SRH, CHARLS used a five-point Likert scale and LSMAEP used a six-point Likert scale. The number of points could have influence respondents’ behavior when assessing whether a “neutral” category existed [19]. In our robustness check, we harmonized the two scales using z-standardization to improve the comparability of the health outcome variable between China and Japan, which was previously used to transform the Likert scale for SRH [20]. The *z*-score was calculated by subtracting the raw score from the mean and dividing the result by the standard deviation.

#### Potential mediators

We considered four variables as potential mediators: low household income, smoking, no leisure-time physical activity (LTPA), and social participation, as suggested in previous studies [6, 7]. We used household spending as a proxy for household income, adjusted for household size by dividing it by the square root of the number of household members [13], and constructed a binary variable for low household income by allocating 1 to the lowest tertile and 0 to the others. For smoking, we constructed a binary variable that indicated current smoking. Regarding LTPA in China, we constructed a binary variable for no LTPA by allocating 1 to respondents who did not engage in LTPA for at least 10 minutes continuously at any level (vigorous, moderate, or light) during a usual week and 0 to others. Based on responses in the LSMAEP, we similarly constructed a binary variable for no LTPA, corresponding to Japanese respondents who did not engage in LTPA at any level during any time period.

Regarding social participation, the CHARLS survey asked respondents whether they participated in 11 types of social activities: (1) interacted with friends; (2) played mahjong, chess, or cards; (3) provided help to non-coresident family, friends, or neighbors; (4) went to sports, social, or other clubs; (5) participated in community-related organizations; (6) volunteer or charitable work; (7) cared for a sick or disabled non-coresident adult; (8) attended an educational or training course; (9) stock investment; (10) used the Internet; and (11) other. For Japan, the LSMAEP asked respondents whether they participated in six types of social activities: (1) hobbies or entertainment, (2) sports or physical exercise, (3) community activities, (4) childcare support or educational or cultural activities, (5) support for the elderly, and (6) others. For both countries, we constructed a binary variable to indicate no social participation (1 = did not participate in any social activity, 0 = had social participation). We did not consider occupational status as a mediator, because most respondents had already retired and were qualified to receive public pension benefits in both countries.

#### Covariates

We considered age and marital status as covariates for both countries by constructing binary variables for each age group from 63 to 72 years and for marital status (1 = married, 0 = unmarried). We also considered residency and *hukou* (household registration) in China by constructing binary variables for urban residency and nonagricultural (urban) *hukou*. *Hukou* determines where people have access to government-provided social services and welfare programs. Residency and *hukou* type represent two systems of urban-rural dichotomy. For instance, many rural-to-urban migrants who work and live in urban cities but still have agricultural *hukou*, are unable to access some social services in the city where they live. Education, employment, health insurance, and medical services are all greatly affected by *hukou*-related urban-rural differences in China, all of which have profound implications for health [21].

### Statistical analysis

We conducted separate statistical analyses for men and women in each country to account for potential sex-based differences in educational inequalities in health, following previous studies [22]. For descriptive analysis, we compared the distribution of educational attainment and the prevalence of poor SRH, low income, smoking, no LTPA, and no social participation between the two countries.

We then conducted a three-step regression and mediation analysis [23, 24]. We initially estimated a logistic regression model (Model 1) to explain the probability of poor SRH using the educational ridit score. The second step was to estimate a set of logistic regression models (Model 2) to explain each of the four potential mediators using the educational ridit score. Third, we estimated a logistic regression model (Model 3) to explain the probability of poor SRH based on educational ridit scores and four potential mediators. In each model, we controlled for covariates and computed the relative index of inequality (RII) [25]. RII corresponds to the estimated odds ratio (OR) of the educational ridit score, which indicates the OR of each outcome from the lowest educational attainment (ridit score = 1) to the highest (ridit score = 0) [26, 27]. In this sense, the conventionally computed OR can be interpreted as RII in this framework.

Finally, we conducted a mediation analysis using the product of coefficients approach to compute indirect effects [24, 28] and estimated the proportion of educational inequalities in health that each mediator could explain. We bootstrapped the results with 2,000 replications to obtain the 95% confidence intervals (CIs) [24].

To check the robustness of the estimation results of the mediation analysis, we conducted two alternative estimations related to the measurement of educational inequality and the construction of the health outcome variable. First, we estimated Models 1 and 3 as linear regression models rather than logistic models, leaving Model 2 unchanged, and computed the slope index of inequality (SII), which corresponds to the estimated coefficient of the educational ridit score. We then conducted a mediation analysis to calculate the proportion of educational inequalities in SRH by each potential mediator. Second, we estimated Models 1 and 3 as linear regression models with z-standardized SRH scores instead of the binary variable of poor SRH and then conducted a mediation analysis with *z*-standardized SRH scores. The Stata software package (version 17) was used for all statistical analyses.

To mitigate potential attrition biases inherited in Japan’s LSMAEP data, we applied the inverse probability weighting method [29, 30]. We first estimated the probit model to explain the probability of participants’ continuation in the survey until the fourteenth wave, factoring in baseline attributes. The inverses of the predicted probabilities were then used as weights in the regression models. Meanwhile, we made no adjustment for China’s CHARLS data, in which a substantial number of new participants were added to each follow-up survey. These new participants were intentionally included to ensure that the CHARLS sample remained representative of the Chinese population aged 45 and above [31].

## Results

### Descriptive analysis

Table 1 summarizes the distribution of educational attainment and ridit scores in China and Japan. In general, Japanese participants had higher educational attainment than their Chinese counterparts. In China, the proportion of those who had graduated from high school or above was 10.3% for men and 4.0% for women, compared with more than 80% for both sexes in Japan, where final educational attainment was heavily concentrated in high school. In both countries, men had higher educational attainment than women. In China, the illiteracy rate among female participants was 44.9%, which was strikingly higher than that of their male counterparts (12.2%). While this illiteracy rate among older Chinese women is surprising, it is consistent with the findings of previous studies [32, 33]. The ridit scores reflect variations in the distribution of educational attainment between men and women, as well as between China and Japan.

**Table 1:** Distribution of educational attainment and ridit scores.

Final educational attainment	Accumulated years of schooling	Men		Women	
		%	Ridit score	%	Ridit score
**China**					
Illiterate	0	12.2	0.939	44.9	0.776
Did not finish primary school	1–5	27.0	0.743	25.6	0.423
Finished primary school	6	29.7	0.460	16.3	0.213
Junior high school	9	20.9	0.207	9.1	0.086
High school and above	12+	10.3	0.051	4.0	0.020
**Japan**					
Junior high school	9	16.6	0.917	16.5	0.917
High school	12	53.2	0.569	64.3	0.513
Junior college	14	2.6	0.290	12.3	0.131
College	16	26.0	0.146	6.8	0.035
Graduate school	18+	1.6	0.008	0.2	0.001

Table 2 compares the prevalence of poor SRH and potential mediators between China and Japan, with the 95% CI of the difference. Chinese participants rated their health as worse than their Japanese counterparts. In contrast to Japan, women in China assessed their health as poorer than their male counterparts did. While the prevalence of smoking among men was more than twice as high in China as in Japan, the prevalence of smoking among women was much lower than that among men and roughly comparable between the two countries. A higher proportion of Japanese participants did not engage in LTPA, whereas a higher proportion of Chinese participants did not participate in any social activity. It should be noted that CHARLS and LSMAEP used different definitions for LTPA and social participation.

**Table 2:** Proportions (%) of each health outcome and potential mediator.

	China	Japan	Difference	
	(A)	(B)	(A)–(B)	95% CI
Men				
Poor self-rated health	27.5	22.6	4.9	(3.0, 6.9)
Low income	32.8	28.6	4.2	(2.2, 6.3)
Smoking	49.1	23.1	26.0	(23.9, 28.1)
No LTPA	9.2	31.7	–22.5	(–24.0, –21.0)
No social participation	54.2	29.1	25.1	(23.0, 27.3)
*Cf*. Married	89.4	86.7	2.7	(1.3, 4.1)
Age (years)	67.0	67.7	–0.7	(–0.8, -0.6)
*N*	2,542	9,221		
Women				
Poor self-rated health	34.8	20.5	14.3	(12.4, 16.2)
Low income	33.9	31.0	2.9	(0.9, 4.9)
Smoking	5.6	5.8	–0.2	(–1.2, 0.8)
No LTPA	9.9	26.0	–16.2	(–17.5, –14.8)
No social participation	51.1	27.6	23.5	(21.4, 25.5)
*Cf*. Married	78.6	77.0	1.6	(–0.1, 3.3)
Age (years)	66.9	67.7	–0.8	(–0.9, –0.7)
*N*	2,735	10,780		

### Regression and mediation analysis

Table 3 summarizes the results of Models 1–3 for men and women in each country. As shown in this table, when we only controlled for covariates in Models 1, RII was 1.69 (95% CI: 1.20–2.39) for men and 1.47 (95% CI: 1.06–2.05) for women in China, compared with 2.70 (95% CI: 2.21–3.28) for men and 2.60 (95% CI: 2.13–3.18) for women in Japan. The results indicated that in both countries, having a lower educational level was associated with a higher risk of poor SRH, but in China, the educational gradient was flatter. Although women in both countries showed somewhat lower educational inequalities in health, the sex difference was not statistically significant. To assess sex-based differences, we included an interaction term between the educational ridit score and females in the regression models for the entire sample. The estimated coefficient of the interaction term was not significant at the 5% significance level (not reported).

**Table 3:** Estimated associations among educational attainment, potential mediators, and self-rated health^a^.

	China				Japan			
	Men		Women		Men		Women	
	OR^b^ (RII^c^)	95% CI^d^	OR (RII)	95% CI	OR (RII)	95% CI	OR (RII)	95% CI
Model 1 [dependent variable = poor self-rated health; independent variable = education ridit score]								
Education ridit score	1.69	(1.20, 2.39)	1.47	(1.06, 2.05)	2.70	(2.21, 3.28)	2.60	(2.13, 3.18)
Models 2 [dependent variable = each mediator; independent variable = education ridit score]								
Low income	1.78	(1.28, 2.49)	2.47	(1.75, 3.48)	2.82	(2.36, 3.36)	3.09	(2.59, 3.67)
Smoking	1.14	(0.84, 1.55)	0.86	(0.46, 1.63)	2.43	(2.00, 2.95)	2.23	(1.57, 3.15)
No LTPA^e^	1.00	(0.59, 1.69)	1.63	(0.96, 2.76)	3.68	(3.08, 4.41)	2.78	(2.31, 3.35)
No social participation	2.38	(1.74, 3.25)	2.42	(1.77, 3.30)	6.70	(5.32, 8.45)	8.96	(7.17, 11.2)
Model 3 [dependent variable = poor self-rated health; independent variables = education ridit score and four mediators]								
Education ridit score	1.68	(1.18, 2.37)	1.37	(0.99, 1.92)	1.99	(1.62, 2.44)	1.83	(1.49, 2.25)
Low income	0.86	(0.70, 1.04)	1.02	(0.86, 1.22)	1.09	(0.97, 1.21)	1.01	(0.91, 1.12)
Smoking	0.76	(0.64, 0.91)	1.12	(0.79, 1.58)	0.87	(0.77, 0.99)	1.25	(1.03, 1.53)
No LTPA	2.01	(1.51, 2.66)	2.26	(1.74, 2.92)	1.38	(1.23, 1.55)	1.61	(1.45, 1.80)
No social participation	1.24	(1.03, 1.49)	1.20	(1.02, 1.41)	2.15	(1.89, 2.44)	1.94	(1.73, 2.19)
*N*	2,542		2,735		9,211		10,780	

^a^ Controlled for age and marital status

^b^ Odds ratio

^c^ Relative index of inequality

^d^ Confidence interval

^e^ Leisure-time physical activity

The Model 2 results indicated that the educational gradient in each potential mediator was generally flatter in China than in Japan. Notably, in sharp contrast to the results for Japan, neither smoking nor LTPA was associated with educational attainment in China. In both countries, however, social participation was positively associated with educational attainment. Another noteworthy finding is that, for both men and women, reductions in the OR of the educational ridit score from Model 1 (which included only the educational ridit score) to Model 3 (which included both the ridit score and potential mediators) were substantially smaller in China than in Japan. We also found that, in China, only social participation was associated with both educational attainment and SRH; whereas in Japan, social participation and LTPA (and smoking for women) were both associated with educational attainment and SRH.

Based on the estimation results in Tables 3 and 4 presents the proportions of educational inequalities in health explained by each potential mediator, along with their bootstrap-estimated 95% CIs. Consistent with the results in Table 3, social participation functioned as a mediator in the association between educational attainment and health in China. However, income, smoking status, and LTPA did not. In China, the proportion of educational inequality partially mediated by social participation was as low as approximately 10% for both sexes. In Japan, LTPA and social participation mediated a substantial portion of educational inequalities in health. Together, these two factors accounted for approximately 37% and 40% of the educational inequalities for men and women, respectively, which is considerably higher than in China. Meanwhile, income did not mediate educational inequalities in health in both China and Japan. Smoking was identified as a mediator only for Japanese women. In both countries, social participation was a key mediator in the effect of educational attainment. Furthermore, we observed that the total proportion of the association mediated by the four mediators was higher in Japan than in China. Additionally, it is noteworthy that the total mediating effect was not significant among Chinese men.

**Table 4:** Estimated proportions (%) of the educational inequalities in self-rated health mediated by each potential mediator^a^.

	China				Japan			
	Men		Women		Men		Women	
	%	95% CI	%	95% CI	%	95% CI	%	95% CI
Low income	–4.4	(–12.7, 0.5)	1.3	(–8.3, 11.6)	2.3	(–0.1, 5.1)	0.4	(–2.5, 3.4)
Smoking	–1.9	(–7.6, 2.0)	–1.2	(–10.4, 2.1)	–2.6	(–5.2, -0.4)	2.0	(0.3, 4.5)
No LTPA^c^	0.0	(–12.8, 11.9)	15.2	(0.0, 35.1)	10.6	(7.1, 14.4)	11.7	(8.6, 15.2)
No social participation	9.5	(1.5, 20.2)	10.2	(1.1, 21.6)	25.9	(21.1, 31.7)	28.5	(22.9, 34.0)
Total	3.3	(–15.0, 19.6)	25.5	(3.3, 49.3)	36.2	(30.3, 43.2)	42.7	(35.9, 49.7)

^a^ Based on results in Table 3.

^b^ Confidence interval estimated by bootstrapping (2,000 replications).

^c^ Leisure-time physical activity

For robustness check, we re-estimated Models 1 and 3 using SII as a measure of educational inequality and z-standardized SRH scores as the dependent variable. Likewise, we repeated the mediation analysis using alternative approaches to measure educational inequality and construct the health outcome variable. The results of these robustness checks, reported in S1 and S2 Tables, are largely in line with those in Tables 3 and 4, both in magnitude and significance, consistent with prior estimates. In addition, considering the observed importance of social participation as a mediator, we computed the *z*-score of the total number of social activities participated for each country, and did mediation analysis by replacing a binary variable of no social participation with a continuous variable of the z-score. The estimation results, which are reported in S3 Table, show that the results remained largely the same as those in Table 4.

## Discussion

Although the scholarly literature on education and health is growing [1–3, 7–12], previous study did not directly compare educational inequalities in general health in China and Japan, nor have they examined potential mediators underlying this association. Given that both countries are experiencing rapid population ageing but have distinct distributions of educational attainment, we examined the association between educational inequalities and SRH in late adulthood. We summarize the key findings and their implications as follows.

First, our study found strong evidence for an educational gradient in health in both China and Japan, which is consistent with most previous studies [1–3, 9, 11]. Unsurprisingly, lower educational levels were associated with a higher risk of poor SRH in both countries.

Second, Japan had a stronger educational gradient in health than China. Given the remarkable concentration of final educational attainment in high school, employers in Japan have been more likely to recognize individuals with a bachelor’s degree or higher. This was especially true during Japan’s economic miracle era, from the end of WWII to the early 1990s, a period marked by the transition of well-educated Japanese people from universities to the workplace. From a life-course perspective, education in Japan could serve as a ladder of upward intra-generational mobility—a social movement from a disadvantaged socioeconomic position to an advantaged one during one’s lifetime [34]. In this process, the better-educated might also benefit from healthier work and living environments, more opportunities for social participation, and more LTPA, which mirrors the findings in Model 2. By contrast, most Chinese participants were not highly educated. After embracing marketization in 1978, China also experienced an economic miracle, but many jobs in the 1980s and 1990s were in low-skilled, labor-intensive sectors such as apparel and textiles. The major industries in Japan are automobiles, consumer electronics, and other types of skilled manufacturing. Therefore, formal education tends to have less influence on social mobility and health in China than in Japan.

It is intriguing to compare our findings in China and Japan with evidence from other countries. One study suggests that differences in socio-political contexts may explain observed educational variations in general health among European countries [35]. For example, in the 1980s, social policies focused on addressing unemployment and income inequalities rather than educational inequality, was proposed to explain why Nordic, egalitarian countries had greater educational variations in morbidity than other European countries [35]. Similarly, our findings of a flatter gradient in China than in Japan may also be explained by historical socioeconomic background, particularly the importance of educational attainment in employment.

It should be noted, however, that the difference in the educational gradient in health between China and Japan was more pronounced among women than among men. As seen in Model 1 results in Table 3, the 95% CI of RII for Chinese women was below that for Japanese women, while 95% CIs of RII for men were overlapped between the two countries. This finding suggests that Chinese women with higher educational attainment can enjoy more limited health benefit probably due to their employment-related issues, compared to the Japanese counterpart.

Third, the influence of potential mediators on educational gradients in health was more limited in China than in Japan. In China, only social participation mediated a small portion (approximately 10%) of educational inequalities; while household income, smoking, or LTPA did not. In Japan, LTPA and social participation together mediated a sizable portion (approximately 30–36%) of educational inequalities in health. After controlling for these mediators, the difference in the RII of poor SRH between the two countries was substantially reduced, suggesting the importance of mediators in explaining discrepancies in educational inequalities in late-life health between the two countries.

Fourth, our results underscore the importance of social participation as a mediator of educational inequality in health in both countries. As suggested by previous studies [7, 36–38], the mediating role of social participation can be explained by the enhanced quality of life, improved psychological well-being, expanded social support, and engaging in more protective health behaviors for staying physically active. The mediating effect of social participation has also been observed in European countries [35], suggesting that social participation may be a context-free, universal mediating factor.

Fifth, however, we observed that the mediating effect of social participation was more substantial in Japan than in China. It is worth noting that CHARLS and LSMAEP defined social participation in different ways. Previous studies suggested that social activities can vary significantly based on cultural and contextual factors and can be further differentiated into caring-based activities (e.g., grandparenting) and productive-based activities (e.g., dancing in public spaces and playing mahjong) [39]. These various types of social activities may indicate different socioeconomic backgrounds and generate differential health implications [39]. Our measures of social participation may capture variations in the caring and productive components of social activities, potentially leading to a greater mediating effect in Japan than China. More specifically, the Japanese list of social activities contained more caring components than the Chinese counterpart. Notably, the Chinese list omitted caregiving for grandchildren or adults in the same household. Hence, the results may suggest that the caring components of social activities may have stronger mediating effects on the association between education and SRH than the productive components.

This study has several limitations, in addition to the possibility of insufficient control for attrition biases. First, the existence of potential cohort effects warrants caution when generalizing results across cohorts. Our sample was restricted to those aged 63–72 years in 2018; however, the distribution of educational attainment may vary among cohorts. In China, for example, the national education system was restored after the Cultural Revolution ended in 1978 and has undergone profound reform since the Compulsory Education Law took effect in 1986 [40]. Second, potential endogeneity was not addressed. Although formal education is normally completed in young adulthood, making simultaneity bias implausible, the causal effects of health status on the mediators cannot be ruled out. For example, healthy individuals are more likely to engage in social activities. Third, other potential mediators, such as health literacy and dieting habits, might exist but were not examined in this study due to a lack of data. Fourth, due to the vastly different distribution of educational levels between the two countries in our sample, we were unable to harmonize the classification of educational levels. Instead, we applied the ridit score to focus on the relative position rather than the absolute level of educational attainment. Fifth, CHARLS and LSMAEP adopted different definitions of social participation with respect to types of social activities and how respondents participated (alone, with other people, or with an organization). The challenge of harmonizing different definitions between countries makes it difficult to interpret the differential magnitudes of the mediating effects of social participation on educational inequalities in health. Sixth, to estimate the indirect effects of educational inequalities on poor SRH via mediators, we imposed necessary assumptions, which included the absence of exposure-outcome, mediator-outcome, exposure-mediator confounders, or mediator-outcome confounder that is affected by the exposure [41]. However, it is impossible to control for all unobservable but plausible confounders, such as all kinds of childhood adversities that may affect both educational attainment and later-life health. Moreover, given the lengthy lag between educational attainment and social participation, the fourth assumption which requires short time between the exposure and the mediator is concerning [41].

## Conclusion

The results underscore that one’s relative position in educational inequalities is a reliable predictor of subjective health in late adulthood in both China and Japan, although the educational gradient was flatter in China. We also found that social participation was a key mediator of educational inequalities in health in both countries. Policy measures to promote social participation are expected to help mitigate educational inequalities in later life health.

## Supporting information

S1 Table Estimated associations among educational attainment, potential mediators, and self-rated health: Alternative approaches. (DOCX)

S2 Table Estimated proportions (%) of the educational inequalities in self-rated health mediated by each potential mediator: Alternative approaches. (DOCX)

S3 Table Estimated proportions (%) of the educational inequalities in self-rated health mediated by each potential mediator: Using the *z*-score of social participation. (DOCX)
