# Supplementary material for: Educational inequalities in self-rated health and their mediators in late adulthood: Comparison of China and Japan
Source: PLoS One. 2023 Sep 15;18(9):e0291661. doi: 10.1371/journal.pone.0291661 (PMC10503706; doi:10.1371/journal.pone.0291661)
Supplement: S1 Table — (DOCX) [file pone.0291661.s001.docx]

**S1 Table. Estimated associations among educational attainment, potential mediators, and self-rated health: alternative approaches**^a^

1. **Dependent variable = poor self-rated health; focusing on the slope index of inequality (SII)**

|  |  | China | | | | | | Japan | | | | | |
| --- | --- | --- | --- | --- | --- | --- | --- | --- | --- | --- | --- | --- | --- |
|  | Independent variable | Men | | | Women | | | Men | | | Women | | |
|  |  | Coef.  (SII) | 95% CI^b^ | | Coef.  (SII) | 95% CI | | Coef.  (SII) | 95% CI | | Coef.  (SII) | 95% CI | |
| Model 1 | |  |  |  |  |  |  |  |  |  |  |  |  |
|  | Education ridit score | 0.10 | (0.04, | 0.17) | 0.09 | (0.01, | 0.16) | 0.18 | (0.14, | 0.21) | 0.16 | (0.13, | 0.19) |
| Model 3 | |  |  |  |  |  |  |  |  |  |  |  |  |
|  | Education ridit score | 0.10 | (0.03, | 0.17) | 0.07 | (0.00, | 0.14) | 0.12 | (0.08, | 0.15) | 0.10 | (0.06, | 0.13) |
|  | Low income | –0.03 | (–0.07, | 0.01) | 0.01 | (–0.03, | 0.04) | 0.01 | (–0.01, | 0.03) | 0.00 | (–0.02, | 0.02) |
|  | Smoking | –0.05 | (–0.09, | -0.02) | 0.03 | (–0.05, | 0.10) | –0.02 | (–0.05, | 0.00) | 0.04 | (0.01, | 0.08) |
|  | No LTPA^c^ | 0.15 | (0.09, | 0.21) | 0.19 | (0.13, | 0.25) | 0.06 | (0.04, | 0.08) | 0.09 | (0.06, | 0.11) |
|  | No social participation | 0.04 | (0.01, | 0.08) | 0.04 | (0.00, | 0.08) | 0.16 | (0.13, | 0.19) | 0.13 | (0.11, | 0.15) |
|  | *N* | 2,542 | | | 2,735 | | | 9,211 | | | 10,780 | | |

1. **Dependent variable = *z* score of self-rated health**

|  |  | China | | | | | | Japan | | | | | |
| --- | --- | --- | --- | --- | --- | --- | --- | --- | --- | --- | --- | --- | --- |
|  | Independent variable | Men | | | Women | | | Men | | | Women | | |
|  |  | Coef. | 95% CI | | Coef. | 95% CI | | Coef. | 95% CI | | Coef. | 95% CI | |
| Model 1 | |  |  |  |  |  |  |  |  |  |  |  |  |
|  | Education ridit score | 0.23 | (0.07, | 0.38) | 0.13 | (–0.01, | 0.26) | 0.51 | (0.42, | 0.59) | 0.42 | (0.34, | 0.50) |
| Model 3 | |  |  |  |  |  |  |  |  |  |  |  |  |
|  | Education ridit score | 0.22 | (0.06, | 0.37) | 0.06 | (–0.08, | 0.20) | 0.36 | (0.27, | 0.44) | 0.26 | (0.18, | 0.34) |
|  | Low income | –0.11 | (–0.20, | –0.02) | 0.04 | (–0.04, | 0.12) | 0.01 | (–0.04, | 0.06) | 0.01 | (–0.03, | 0.05) |
|  | Smoking | –0.07 | (–0.15, | 0.01) | 0.08 | (–0.08, | 0.24) | –0.05 | (–0.10, | 0.00) | 0.07 | (–0.01, | 0.15) |
|  | No LTPA | 0.20 | (0.06, | 0.33) | 0.29 | (0.17, | 0.41) | 0.18 | (0.13, | 0.23) | 0.21 | (0.16, | 0.26) |
|  | No social participation | 0.12 | (0.04, | 0.20) | 0.12 | (0.04, | 0.19) | 0.40 | (0.34, | 0.47) | 0.34 | (0.28, | 0.39) |
|  | *N* | 2,542 | | | 2,735 | | | 9,211 | | | 10,780 | | |

^a^ Controlled for ages and marital status. Models 1 and 3 were linear regression models, and the results of Model 2 (logistic models) results were the same as those in Table 3 and not reported. The slope index of inequality (SII) corresponds to the estimated coefficient of the educational ridit score.

^b^ Confidence interval

^c^ No leisure-time physical activity
