# Supplementary material for: Educational inequalities in self-rated health and their mediators in late adulthood: Comparison of China and Japan
Source: PLoS One. 2023 Sep 15;18(9):e0291661. doi: 10.1371/journal.pone.0291661 (PMC10503706; doi:10.1371/journal.pone.0291661)
Supplement: S2 Table — (DOCX) [file pone.0291661.s002.docx]

**S2 Table.** **Estimated proportions (%) of the educational inequalities in self-rated health mediated by each potential mediator: alternative approaches**^a^

1. **Dependent variable = poor self-rated health; focusing on the slope index of inequality (SII)**

|  | China | | | | | | Japan | | | | | |
| --- | --- | --- | --- | --- | --- | --- | --- | --- | --- | --- | --- | --- |
|  | Men | | | Women | | | Men | | | Women | | |
|  | % | 95% CI^b^ | | % | 95% CI | | % | 95% CI | | % | 95% CI | |
| Low income | –4.4 | (–12.6, | 0.5) | 1.3 | (–7.6, | 11.8) | 2.3 | (–0.1, | 5.1) | 0.4 | (–2.5, | 3.4) |
| Smoking | –1.8 | (–7.5, | 2.1) | –1.3 | (–11.6, | 1.9) | –2.6 | (–5.2, | -0.4) | 2.0 | (0.3, | 4.5) |
| No LTPA^c^ | –0.1 | (–12.5, | 15.4) | 16.5 | (0.0, | 40.3) | 10.6 | (7.1, | 14.4) | 11.7 | (8.6, | 15.2) |
| No social participation | 9.3 | (1.5, | 19.6) | 10.1 | (1.3, | 22.0) | 25.9 | (21.1, | 31.7) | 28.5 | (22.9, | 34.0) |
| Total | 3.0 | (–14.3, | 22.7) | 26.6 | (4.2, | 53.4) | 36.2 | (30.3, | 43.2) | 42.7 | (35.9, | 49.7) |

1. **Dependent variable = *z* score of self-rated health**

|  | China | | | | | | Japan | | | | | |
| --- | --- | --- | --- | --- | --- | --- | --- | --- | --- | --- | --- | --- |
|  | Men | | | Women | | | Men | | | Women | | |
|  | % | 95% CI | | % | 95% CI | | % | 95% CI | | % | 95% CI | |
| Low income | –7.3 | (–16.8, | -1.9) | 1.8 | (–11.5, | 15.3) | 2.2 | (–0.4, | 5.0) | 0.3 | (–2.4, | 3.2) |
| Smoking | –1.1 | (–6.5, | 0.8) | –2.0 | (–17.8, | 2.5) | –2.6 | (–4.9, | –0.3) | 2.2 | (0.3, | 4.9) |
| No LTPA | 0.0 | (–8.6, | 9.6) | 15.0 | (–0.9, | 38.1) | 10.6 | (7.0, | 14.4) | 12.0 | (8.5, | 15.7) |
| No social participation | 12.5 | (4.7, | 24.9) | 16.6 | (4.9, | 32.1) | 29.9 | (23.8, | 36.5) | 32.4 | (25.4, | 39.4) |
| Total | 4.1 | (–10.3, | 19.3) | 31.4 | (4.2, | 60.5) | 40.1 | (33.0, | 47.9) | 47.0 | (39.0, | 55.2) |

^a^ Based on results in Table S1.

^b^ Confidence interval estimated by bootstrapping (2,000 replications).

^c^ No leisure-time physical activity
