# Supplementary material for: Educational inequalities in self-rated health and their mediators in late adulthood: Comparison of China and Japan
Source: PLoS One. 2023 Sep 15;18(9):e0291661. doi: 10.1371/journal.pone.0291661 (PMC10503706; doi:10.1371/journal.pone.0291661)
Supplement: S3 Table — (DOCX) [file pone.0291661.s003.docx]

**S3 Table.** **Estimated proportions (%) of the educational inequalities in self-rated health mediated by each potential mediator: using the z-score of social participation**

|  | China | | | | | | Japan | | | | | |
| --- | --- | --- | --- | --- | --- | --- | --- | --- | --- | --- | --- | --- |
|  | Men | | | Women | | | Men | | | Women | | |
|  | % | 95% CI^b^ | | % | 95% CI | | % | 95% CI | | % | 95% CI | |
| Low income | –4.4 | (–12.5, | 0.6) | 0.6 | (–9.3, | 10.8) | 2.3 | (–0.6, | 5.1) | 0.4 | (–2.6, | 3.6) |
| Smoking | –1.9 | (–8.1, | 2.2) | –1.2 | (–11.5, | 1.9) | –3.2 | (–6.2, | -0.9) | 2.2 | (0.4, | 4.9) |
| No LTPA^c^ | 0.0 | (–11.4, | 11.6) | 14.9 | (–1.3, | 33.9) | 9.9 | (6.2, | 14.0) | 12.5 | (9.1, | 16.1) |
| *z*-score of social participation | 13.6 | (1.4, | 27.8) | 18.5 | (7.4, | 33.5) | 26.4 | (21.4, | 31.8) | 30.3 | (24.7, | 36.8) |
| Total | 7.3 | (–12.7, | 24.9) | 32.8 | (10.2, | 57.9) | 35.5 | (29.1, | 42.2) | 45.4 | (38.4, | 53.4) |

^a^ Based on results in Table S1.

^b^ Confidence interval estimated by bootstrapping (2,000 replications).

^c^ No leisure-time physical activity
